# From Individual to Stand Performance in Hybrids: Challenging the Optimal Parental Genetic Distance

**DOI:** 10.1111/eva.70165

**Published:** 2025-10-09

**Authors:** Catharina Y. Utami, Cyrille Violle, Denis Vile, Lisa Perrier, François Vasseur

**Affiliations:** ^1^ CEFE Univ Montpellier, CNRS, EPHE, IRD Montpellier France; ^2^ LEPSE Univ Montpellier, INRAE, Institut Agro Montpellier France

**Keywords:** *Arabidopsis thaliana*, F2 population, genetic distance, hybrid performance, plant interactions, transgressive segregation

## Abstract

Hybridization, the interbreeding of distinct genotypes, drives evolutionary processes like speciation and adaptation, potentially via phenotypic transgression, where hybrids exhibit novel traits. In crop breeding, research has largely focused on optimizing heterosis to enhance hybrid performance, particularly for traits such as biomass. It is only recently that the ecological implications of hybridization have been considered, highlighting hybridization as a biotic interaction occurring within populations and communities. This shift raises fundamental questions about whether hybrid performance shows consistent patterns across individual and population scales, particularly regarding predictions based on parental genetic distance. Here, we address this question by examining *Arabidopsis thaliana* F2 hybrids across a wide range of genetic distances, to compare hybrid performance at individual and stand levels. Our results reveal scale‐dependent patterns: individual performance peaks at intermediate parental genetic distances, while stand‐level performance increases with genetic divergence, particularly in hybrids between relict and non‐relict lineages. These results underscore the importance of scale when evaluating hybrid performance, as plant–plant interactions at the group level can alter the collective outcomes of individual performance. Finally, this framework underscores the importance of integrating individual and population perspectives to better understand the outcomes and potential applications of hybridization.

## 1 Introduction

Hybridization, the interbreeding of divergent genotypes (Anderson and Stebbins Jr. 1954), whether within species (intraspecific) or between species (interspecific), has captivated biologists for generations. Charles Darwin, in his seminal work On the Origin of Species, recognized hybridization as a natural process that generates variation, a fundamental element of natural selection (Livezey and Darwin 1953). Study on hybridization has since moved forward, widely extending to agricultural perspectives, where artificial hybridization is often generated to maximize crop yield and production (Cheres et al. 2000; Jiang et al. 2017; Zhao et al. 2015). Either through natural or artificial means, hybridization represents the same fundamental process, altering organismal performance relative to their parental lines and often leading to changes in traits such as productivity, adaptability, or fitness. Despite considerable advancements in understanding hybridization's role within both evolutionary and agricultural frameworks, only recently has research begun to address its ecological implications. Hybridization can be viewed as a biotic interaction, as suggested by (Porretta and Canestrelli 2023), occurring within the context of ecological populations and communities. While hybridization begins at the cellular and individual level, its effects extend to population dynamics, where biotic interactions such as competition come into play.

In nature, plants rarely grow in isolation. Neighboring plants strongly influence one another's success and, consequently, group performance (Milbau et al. 2007), highlighting the role of plant–plant interactions, such as competitive pressure, in shaping group dynamics. Research has shown that plant fitness can differ between individual and group scales under competitive conditions (Wuest et al. 2022). In hybrid populations, F1 individuals typically produce seeds that disperse locally, leading to F2 populations of closely related individuals competing in proximity. Thus, hybridization forms populations where intraspecific competition is highly localized, particularly in herbaceous species with limited dispersal. In the F2 generation, recombination introduces segregation variance, leading to increased genetic diversity (Lande 1981). This heightened genetic variation in F2 hybrids often leads to transgressive segregation, resulting in novel phenotypes extending beyond the parental range (Palacio‐López and Molofsky 2021). Such genetic and phenotypic diversity may enable hybrids to exploit a broader range of ecological niches, reduce resource overlap, and influence population dynamics (Gross et al. 2009; Violle et al. 2012). However, the role of genetic diversity in shaping community‐level performance remains unclear and requires further investigation, as studies often report conflicting results regarding its role in productivity (Huber et al. 2016; Hughes et al. 2008). Testing hybrid performance across different densities and between different genotypes is essential to understanding how plant–plant interactions influence hybridization outcomes. One promising approach is through explicit experimental comparisons of individual and stand‐level performance, yet such studies remain surprisingly rare (but see studies on multilevel selection at group and individual scales Goodnight (1985)); (Goodnight and Kalisz 1995). To our knowledge, no empirical studies have yet examined this question in hybrid populations, leaving a critical gap in understanding whether genetic distance effects manifest differently across organizational levels in such systems.

The relationship between genetic distance and hybrid performance has been extensively studied in artificial hybrids to exploit hybrid vigor (heterosis) that can enhance crop productivity. In such contexts, research often focuses on selecting parental combinations or genetic distances that generate the most productive individuals (Barth et al. 2003; Groszmann et al. 2014; Oakley et al. 2015). For instance, a key question is whether hybrid performance increases (linearly) with parental genetic distance, or whether there is specific optimal mating distance due to balance between heterosis and inbreeding‐outbreeding depression (Cheres et al. 2000; Palacio‐Lopez et al. 2018; Stuber et al. 1992; Wei and Zhang 2018; Würschum et al. 2023). Some have failed to demonstrate an optimal mating distance (Robinson et al. 2009; Yang et al. 2017), and found a positive correlation instead (Reif et al. 2003). In contrast, there are some experimental supports on the optimal mating distance, with research across various taxa showing that intermediate genetic distance often leads to improved hybrid performance for specific traits (Palacio‐Lopez et al. 2018; Rajan et al. 2023; Wei and Zhang 2018). Studies on heterosis often focus on the F1 generation, where maximum heterosis and uniformity are observed, though these effects persist to a lesser extent in the F2 and subsequent generations (Greaves et al. 2015; Wu et al. 2004). For instance, F2 hybrids of *Arabidopsis thaliana* are shown to exhibit significant heterosis for traits such as biomass yield and rosette diameter (Barth et al. 2003). Moreover, enhanced trait variation due to recombination in F2s may modulate plant–plant interactions, such as niche complementarity, and thus modify the relationship between parental distance and hybrid performance. Determining the contribution of heterosis and phenotypic segregation to the performance of hybrid populations is critical to understand the evolutionary consequences of hybridization. Studies using F2 hybrids have explored the effects of genetic diversity on performance, demonstrating that correlations can vary depending on traits and environmental conditions (Gutiérrez et al. 2002). However, no studies have yet examined how genetic distance affects both individual and group‐level hybrid performance or explored the role of ancestry group combinations in shaping these outcomes. Filling this gap is crucial for advancing our understanding of hybrid dynamics across scales.

*A. thaliana* has long served as a model organism for plant genetics and molecular biology. More recently, it has become a focus for ecologists and evolutionary biologists, following the availability of large‐scale genomic datasets from natural accessions across its range (Alonso‐Blanco et al. 2016; Horton et al. 2012; Weigel 2012). These datasets have shed light on the recent demographic history of the species, revealing two major lineages. Most natural lines in Europe belong to non‐relict lineages, which arose from a secondary colonization event in central Europe approximately 10,000 years ago. In contrast, relict lineages diverged during the Ice Age and have remained highly isolated, adapting to distinct ecological niches. Relict populations are typically found in ancient oak and pine forests characterized by seasonal climates, high temperatures, and low rainfall, whereas non‐relicts are often associated with human‐modified habitats like agricultural and urban areas (Alonso‐Blanco et al. 2016). Interestingly, hybridization events between relict and non‐relict lineages of *A. thaliana* have been documented in northern and southern Europe, which has been hypothesized to contribute to local adaptations (Exposito‐Alonso et al. 2018; François et al. 2008; Hanemian et al. 2020; Lee et al. 2017). However, it is still unclear whether the maintenance of natural hybrids in some regions has been facilitated by the consequences of hybridization on population performance.

In this study, we examined hybrid performance at individual and stand levels in *A. thaliana* , focusing on whether the relationship between parental genetic distance and hybrid performance varied across scales. Experiments were conducted on 63 F2 hybrids, representing a range of parental genetic and phenotypic distances. We also investigated whether specific lineage or ancestry combinations influenced hybrid performance. Biomass production was measured at both individual and stand levels to assess how genetic distance and lineage effects observed at the individual scale translated to population‐level contexts.

## 2 Materials and Methods

### 2.1 Plant Material

**FIGURE 1:**
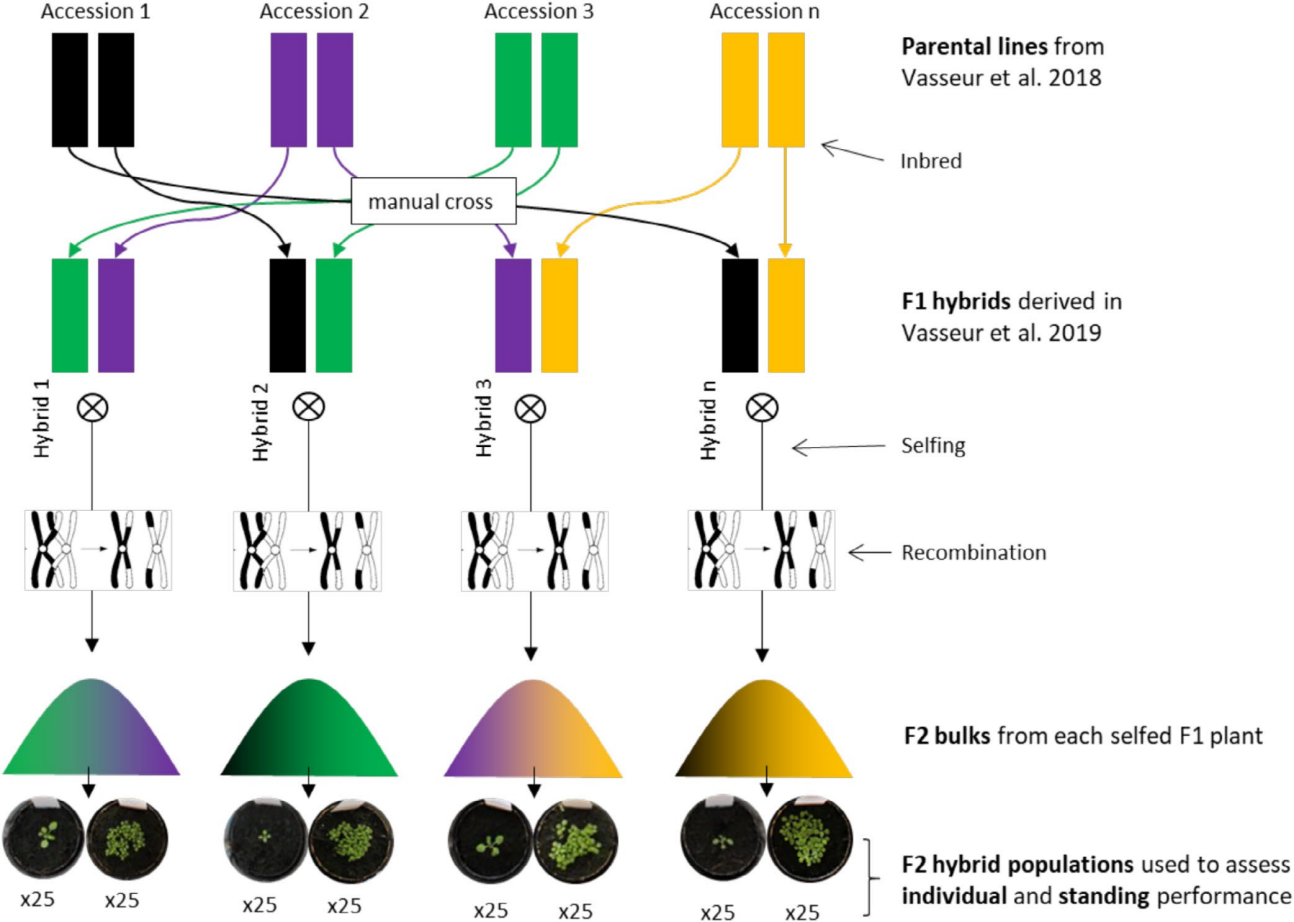
Schematic overview of the derivation of F2 hybrid populations and experimental design used in this study. Parental lines were selected from the natural accessions characterized by the 1001 Genomes Consortium (2016), and previously used in Vasseur et al. (2018). These accessions represent a broad gradient of genetic distances and belong to distinct admixture groups as defined in the 1001 Genomes dataset. F1 hybrids were generated through manual crosses (Vasseur et al. 2019) and subsequently self‐fertilized to produce F2 seeds. From these, 63 F2 hybrid populations were chosen to maximize coverage of the parental genetic distance gradient. Bell curves with color gradation illustrate the expected variability in F2 bulks due to segregation and recombination. For each hybrid, seeds were randomly drawn to produce 25 pots for assessing individual biomass and 25 pots for assessing stand biomass, with each stand sample consisting of multiple seeds.

To investigate how genetic distance influences hybrid performance, we selected 63 F2 hybrid populations that represent a broad spectrum of pairwise genetic distances (Table S1). These populations were chosen based on genetic distance data from prior studies (Vasseur et al. 2018; Vasseur et al. 2019) which also provided the F2 seeds. The hybrids were derived from 97 natural *A. thaliana* accessions, which span a diverse range of geographic origins and phenotypic traits (biomass) (Przybylska et al. 2023); (Figures S1 and S2). This design ensures that the selected hybrids capture variation in genetic distance across different spatial contexts. In the original studies, intraspecific F1 hybrids were created through manual crosses between inbred natural accessions, and the resulting F1 plants were selfed to produce F2 populations (Figure 1). Parental genetic distances were quantified as the number of allelic differences, using PLINK's‐distance function, based on a filtered dataset of 391,016 SNPs (Exposito‐Alonso et al. 2018).

To further explore the role of lineage in hybrid performance, we utilized the ancestry classifications from the 1001 Genomes Consortium project. Accessions were grouped into relict and non‐relict lineages, with the non‐relict group further divided into subgroups: North Sweden, South Sweden, Germany, Asia, Western Europe, Admixed, Central Europe, Spain, and Italy–Balkans–Caucasus (Alonso‐Blanco et al. 2016). We classified the hybrids into three categories based on these lineage groups: 1. hybrids between relict and non‐relict lineages (inter‐relict); 2. hybrids between different ancestral groups of the non‐relict lineage (inter‐group); and 3. hybrids between the same ancestral group of the non‐relict lineage (intra‐group). We selected 10 pairs for the inter‐relict group, 44 pairs for the inter‐group, and 9 pairs for the intra‐group.

### 2.2 Growth Conditions

All plants were grown in a greenhouse, located at the Center for Functional and Evolutionary Ecology (CEFE), Montpellier, France (Figure S3). Seeds were sown in 5‐cm pots (80 mL) filled with peat moss (Neuhaus N2) media, in March 2023. Each F2 hybrid population was represented by 25 individual pots, each containing a single plant grown in isolation, and 25 stand pots, where 30 seeds were initially sown (comprising random individuals from the F2 bulk, Figure 1). This setup resulted in 3150 pots across both treatments. Experimental blocks were divided by treatment, and pots within each block were randomly positioned across multiple tables. Each treatment was grown under the same controlled conditions, with stable room temperature (±20°C day/15°C night) and under well‐watered regular irrigation (every 4–5 days).

### 2.3 Trait Measurement

F2 hybrid performance was assessed by measuring aboveground dry mass (biomass). We harvested the plants at the same time after a growth period of approximately 60 days, during early to mid‐May 2023. Plants were harvested in a random order, prioritizing completion before flowering whenever possible. For each pot, plants were cut at ground level, with rosettes and stems separated for those that had reached the reproductive stage. Rosettes were dried at 60°C for at least 48 h before weighing. At the stand level, the number of individuals alive at harvest was recorded. We excluded stands with fewer than 15 plants per pot, resulting in a final dataset of 2126 observations. Stand‐level biomass was standardized to 25 individuals, i.e., the total biomass of all plants in the pot was divided by the number of individuals alive at harvest, then multiplied by 25 (the mean number of individuals alive at harvest).

Trait variation within hybrid populations was quantified using the variance ($S_{n}^{2}$) of individual biomass, calculated as the arithmetic mean of the squared deviations from the mean biomass value:

$$S_{n}^{2}=\frac{1}{n-1}\;\sum_{i=1}^{n}\left(x_{i}-\bar{x}\right)2$$

Measurement of F1 heterosis was obtained using data from Vasseur et al. (2019), and we used their measurements of mid‐parent heterosis (MPH) for vegetative dry mass. MPH is a commonly used metric to quantify heterosis (Seymour et al. 2016) and was calculated for the biomass trait Y, based on the deviation of the observed hybrid value relative to the mean parental value:

$$\mathrm{MPH}=\left(Y_{1\;x\;2}–\text{mean}\left(Y_{1},Y_{2}\right)\right)/\text{mean}\left(Y_{1},Y_{2}\right)$$

To assess phenological variation (asynchrony), we used two proxies: 1. the proportion of flowering individuals within each hybrid population and; 2. the difference in parental flowering time, based on data from a previous study (Vasseur et al. 2018). From the same dataset and from (Przybylska et al. 2023), we also quantified parental trait differences, including vegetative dry mass, flowering time, relative growth rate (RGR), leaf area (LA), leaf dry matter content (LDMC), and root allocation.

### 2.4 Statistical Analysis

To test the relationship between parental genetic distance and hybrid performance, we initially included table positions as a random effect in a mixed model to account for potential block effects. However, the Akaike Information Criterion (AIC) indicated no significant improvement from adding the block effect (AIC difference = 0.447). A likelihood ratio test (LRT) using the logLik() function also yielded a *p* > 0.05, confirming that block effects were negligible. Accordingly, we used generalized linear models (GLMs) or linear models (LMs) instead of mixed models. The relationship between parental genetic distance and individual biomass was analyzed using a GLM with a Gamma distribution, while other relationships were analyzed using linear and polynomial regression. Prior to the regressions, stand biomass, mean biomass, and biomass variance were log10‐transformed to ensure normally distributed residuals.

To choose the best model, linear and polynomial regression models were compared based on Akaike's Information Criterion (AIC). Factors influencing stand performance, such as individual biomass variance and F1 heterosis, were evaluated using multiple regression models. Additionally, hybrid differences based on parental ancestry group combinations were assessed using analysis of variance (ANOVA), with Tukey's post hoc tests performed for group comparisons using the Multcomp package (Hothorn et al. 2008). All statistical analyses were conducted in R (version 4.3.1).

## 3 Results

### 3.1 Effect of Parental Genetic Distance on the Performance of F2 Hybrid Population at Individual and Stand Levels

**FIGURE 2:**
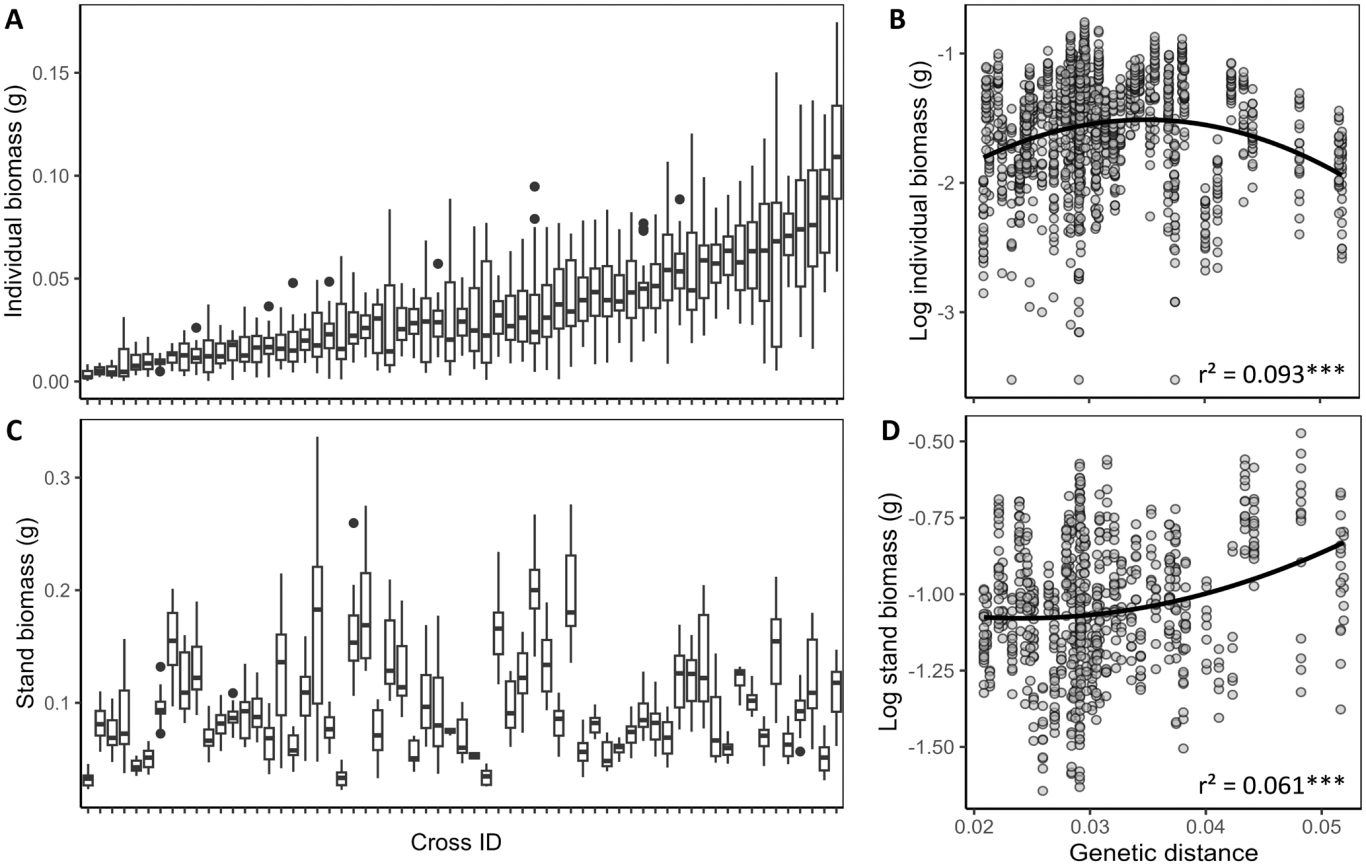
Variation of biomass in F2 hybrids at individual and stand levels and the relationship with genetic distance. Boxplot showing variation of individual biomass (A), and variation of stand biomass (B) across 63 F2 hybrid populations (ANOVA, *p* < 0.001). Number of plants per population ranges from 14 to 25. The relationship between genetic distance and individual biomass (C) supports a humped‐back model. Polynomial regression of the relationship between stand biomass and genetic distance (D) shows deviation from the humped‐back model. *** denotes *p* < 0.001.

Biomass was highly variable across F2 hybrid populations at both individual and stand levels (Figure 2A,C). Mean individual biomass per F2 hybrid population ranged from 0.003 to 0.112 g, while mean stand biomass varied between 0.032 and 0.202 g. Although performance variability was observed at both levels, we found a lack of correlation between individual and stand‐level biomass across F2 hybrid populations (Spearman's *p* = 0.069, *p* = 0.589), indicating a mismatch in the biomass variation pattern between the two levels.

Analysis of the relationship between parental genetic distance and biomass revealed distinct trends for individual and stand‐level outcomes. At the individual level, we observed a hump‐shaped relationship (*r* ^2^ = 0.093, *p* < 0.001): biomass peaked at an intermediate parental genetic distance (Figure 2B). However, at the stand level, we found a polynomial relationship (*r* ^2^ = 0.061, *p* < 0.001): greater genetic distances were associated with increased stand biomass (Figure 2D).

### 3.2 Heterosis and Trait Variation Modulate Stand Performance in F2 Hybrid Populations

**FIGURE 3:**
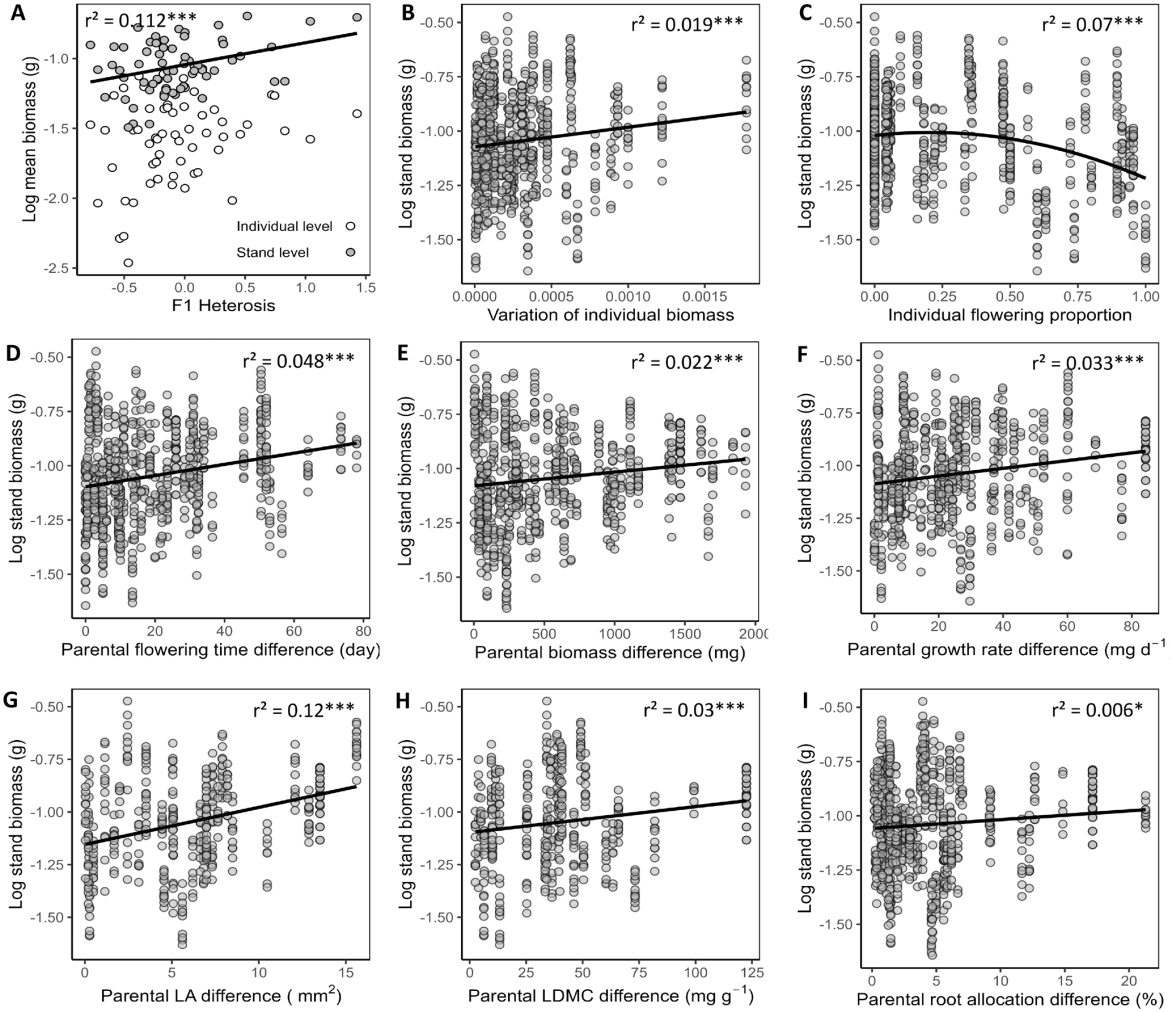
Factors influencing hybrid stand performance. (A) Heterosis enhances hybrid stand biomass but does not affect individual biomass. (B‐C) Greater trait variation in biomass and flowering time increases hybrid stand biomass. (D‐I) Hybrid stand biomass is positively associated with parental trait differences in flowering time, biomass, relative growth rate, leaf area (LA), leaf dry matter content (LDMC), and root allocation. *** denotes *p* < 0.001; * denotes *p* < 0.05.

Using previously published data (Vasseur et al. 2019), we compared mean F2 biomass and F1 heterosis on biomass. We found that F1 heterosis was positively correlated with F2 stand biomass (Figure 3A; *r* ^2^ = 0.112, *p* < 0.01) but not with individual biomass (*r* ^2^ = 0.034, *p* = 0.08). Within F2 populations, greater trait variance among individuals was associated with higher stand biomass (Figure 3B; *r* ^2^ = 0.019, *p* < 0.001). Variation in phenology also influenced stand performance, as we found stand biomass following a significant quadratic relationship with the proportion of flowering individuals, peaking when approximately 50% of individuals were flowering (Figure 3C; *r* ^2^ = 0.07, *p* < 0.001). Additionally, parental flowering time differences positively correlated with stand biomass (Figure 3D; *r* ^2^ = 0.048, *p* < 0.001). Other parental trait differences, including vegetative biomass, relative growth rate, LA, LDMC, and root allocation, also showed significant positive relationships with stand biomass (Figure 3E–I; *r* ^2^ = 0.022, *r* ^2^ = 0.033, *r* ^2^ = 0.12, *r* ^2^ = 0.03, *r* ^2^ = 0.006, respectively; *p* < 0.001 for all traits except root allocation, *p* < 0.05).

### 3.3 Hybridization Between Distinct Lineages Optimizes Stand Performance

**FIGURE 4:**
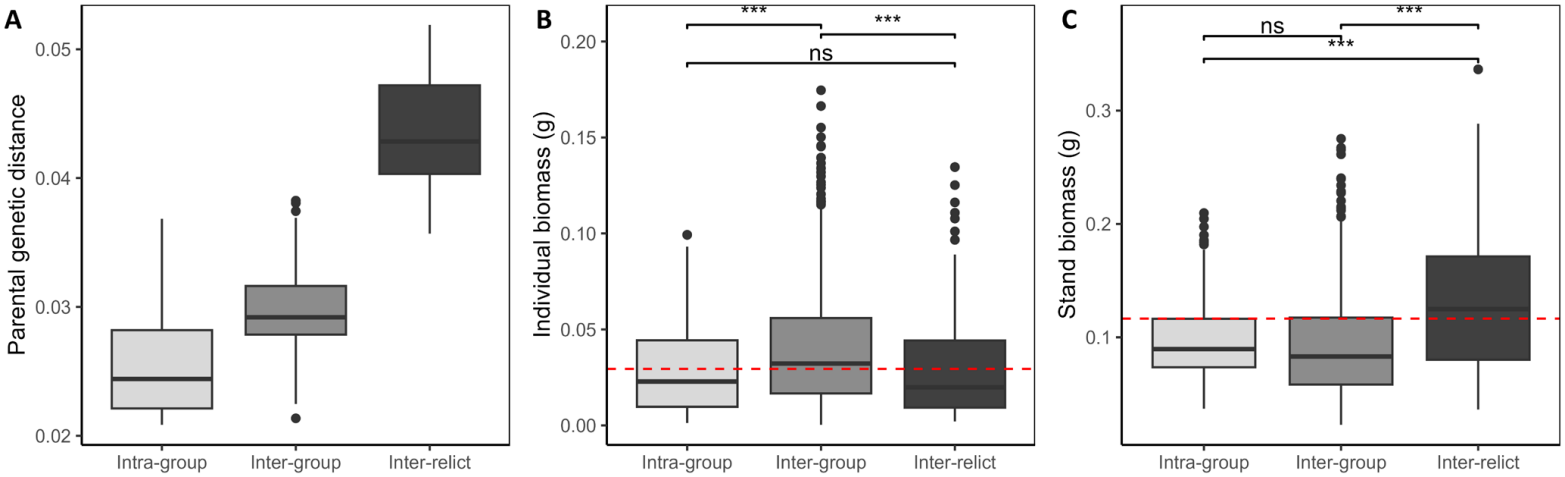
Variation in genetic distance, individual performance, and stand‐level performance across hybrid groups based on different parental ancestry combinations. (A) Boxplot showing parental genetic distance across ancestry groups, with hybrids between different lineages exhibiting greater genetic divergence; (B) boxplot showing individual biomass for each hybrid group; and (C) stand‐level biomass for each group. The red dashed line in panels B and C marks the mean biomass of the inter‐relict group for reference. Here, the “inter‐relict group” includes hybrids between relict and non‐relict lineages, the “inter‐group” hybrids are derived from different ancestral groups within the non‐relict lineages, and the “intra‐group” hybrids originates from the same ancestral group within the non‐relict lineage.

As it has been reported that hybrids between relict and non‐relict lineages have been naturally selected in *A. thaliana* (Exposito‐Alonso et al. 2018; François et al. 2008; Lee et al. 2017), we investigated further how F2 hybrid performance varied in F2 populations derived from crosses between these lineages. Based on lineage composition, we classified hybrids into three groups: inter‐relict, inter‐group, and intra‐group (see Materials and Methods). As previously shown in Alonso‐Blanco et al. 2016, inter‐relict hybrids displayed greater parental genetic distance (Figure 4A). Inter‐group hybrids, which resulted from crosses between different groups of non‐relict accessions, exhibited intermediate genetic distances, while intra‐group hybrids, derived from parents within the same non‐relict lineage, showed the smallest genetic distances.

When comparing individual biomass across these groups, inter‐group hybrids displayed the highest individual biomass, with no significant difference observed between the intra‐group and inter‐relict hybrids (Figure 4B). At the stand level, hybrids formed between relict and non‐relict lineages showed greater stand biomass compared to hybrids solely within non‐relict lineages (Figure 4C). This suggests that crossing distinct lineages enhances overall stand performance relative to hybrids within the same lineage, as stand biomass did not differ significantly between inter‐group and intra‐group hybrids.

## 4 Discussion

The extent to which hybrid performance varies with genetic distance remains elusive. While most studies focus on individual‐level F1 performance, hybridization is a multifaceted process influenced by ecological and genetic contexts that can lead to diverse outcomes. In plants, especially in species with limited dispersal, hybrid populations tend to form over successive generations in the same locality, where plant–plant interactions, such as competition and cooperation, strongly influence hybrid population success. Consequently, generalizing the benefits or risks of hybridization from individual‐level studies alone can overlook the broader ecological and evolutionary dynamics at play. Our findings highlight the scale‐dependent nature of F2 hybrid performance, with individual and stand‐level biomass outcomes responding differently to parental genetic distance.

Research often reports a positive correlation between heterosis and genetic distance, although the strength of this relationship varies. According to Dobzhansky–Muller incompatibility theory, hybrids between species or populations with high genetic divergence are particularly susceptible to outbreeding depression (Lynch 1991; Orr and Turelli 2001), driven by hybrid breakdown in the F2 generation when specific allele combinations reduce individual fitness. Subsequently, several empirical studies have reported a hump‐shaped relationship between genetic distance and hybrid performance, described as the *optimal mating distance* hypothesis (Orr and Turelli 2001; Wei and Zhang 2018). This hypothesis proposes that hybrid vigor is optimized at intermediate genetic distances, where genetic dissimilarity reduces inbreeding depression without introducing severe genetic incompatibilities. Our data at the individual level align with this hypothesis, with individual performance in F2 individuals peaking at intermediate genetic distances. Although the precise mechanisms behind hybrid vigor remain uncertain, they may involve dominance, overdominance, or positive intergenic epistasis (Lippman and Zamir 2007). Supporting this, a study in *A. thaliana* by Oakley et al. (2015) suggested that the hump‐shaped relationship between hybrid performance and mating distance results from a combination of dominance‐based heterosis, which increases linearly with genetic distance, and intergenic interactions like overdominance and epistasis, which exhibit a quadratic relationship with genetic distance.

The Dobzhansky‐Muller model of hybrid incompatibility predicts that mutations segregating in F2 hybrids contribute to hybrid breakdown, leading to both fitness declines and increased phenotypic variation (Koski et al. 2022). While their findings supported this prediction, their analysis focused solely on individual fitness, without accounting for population‐level dynamics or plant–plant interactions. In contrast, our stand‐level analysis reveals that biomass in F2 hybrids increases with genetic distance. Both theoretical and empirical studies indicate that isolated lines or populations often exhibit strong F1 heterosis, followed by a decline in fitness in subsequent generations, as depression typically emerges only after recombination disrupts gene complexes in the F2 or higher‐order hybrid generations (Lynch 1998). The lack of a significant F1 heterosis effect on individual biomass in F2 hybrids may therefore result from increased phenotypic variance within the F2 population.

The role of genetic diversity in shaping community biomass remains a subject of debate in biodiversity–ecosystem functioning (BEF) studies, with many studies reporting both positive and negative effects of species or genotype richness on community performance (Crutsinger et al. 2006; Huber et al. 2016; Pillai and Gouhier 2019; Tilman et al. 2001). This ongoing debate highlights the need to test hybrid performance at different densities and across diverse genotype pairings to disentangle the effects of plant–plant interactions during hybridization. Since different crosses generate varying levels of genotypic and phenotypic diversity, understanding how these variations influence biomass at both individual and stand levels will provide deeper insights into the ecological and evolutionary consequences of hybridization.

Our findings suggest that phenotypic variance between F2 individuals (through biomass and flowering time) had a positive effect on stand biomass, supporting the idea of niche partitioning between hybrids originating from the same parent and locally competing for resources, similar to that observed between species (Hughes and Stachowicz 2004). This phenotypic variance results from recombination during meiosis, which shapes allelic diversity in hybrid progeny (Salomé et al. 2012) found that recombination rates in *A. thaliana* do not correlate with whole‐genome sequence divergence between parental accessions. Accordingly, Koski et al. (2022) observed that transgressive phenotypes, traits that exceed the parental range, appear across all hybrid cross types, with genetic distance not directly influencing the degree of segregation variance. This likely explains why, in our analysis, genetic distance did not correlate with variance in individual hybrid traits. However, when transgressive segregation was assessed across multidimensional traits, such as overall phenotypic range, higher genetic distances were associated with broader phenotypic variation (Stelkens et al. 2009). Additionally, (Benowicz et al. 2020) demonstrated that, although wide‐cross hybrids in F2 generations may not markedly increase segregation variance in individual traits, they can enhance the diversity of trait combinations, potentially expanding the adaptive potential of hybrid populations. Previous research has also shown that hybridization can lead to trait combinations enhancing fecundity and competitiveness, sometimes enabling hybrids to invade new habitats due to their superior “weediness” (Ellstrand and Schierenbeck 2006; Hauser et al. 2003). Recent research highlights trait distinctiveness as a dimension of functional diversity, characterizing the unique role of an individual plant within a competitive group (Mahaut et al. 2023). Trait distinctiveness has been shown to positively affect mixture performance, potentially through mechanisms similar to those observed in transgressive hybrids. Expanding beyond traits measured within the F2 population, we found that phenotypic variation correlated with differences between parental traits (Table S2). We then analyzed how differences in parental traits, including flowering time, biomass, relative growth rate, leaf area (LA), leaf dry matter content (LDMC), and root allocation, affected stand biomass. Our results revealed that greater trait divergence between parents was positively associated with higher F2 hybrid stand biomass. This suggests that increased parental functional trait divergence contributes to a broader range of phenotypic variation in F2 hybrids, potentially enhancing stand‐level performance through niche complementarity.

Phenological asynchrony may be one of the mechanisms contributing to hybrid stand performance. Plant populations naturally exhibit interindividual variation in phenology (Ehrlén and Münzbergová 2009), and this variation has a genetic basis (Wilczek et al. 2009). While some studies suggest that phenological synchronization enhances reproductive success by facilitating pollination and seed set (Bogdziewicz et al. 2020; Ims 1990; Rodríguez‐Pérez and Traveset 2016), others indicate that phenological variation within a population can reduce intraspecific competition and enhance biomass accumulation (Elzinga et al. 2007; Fogelström and Ehrlén 2019; Kang et al. 2024; Munguía‐Rosas et al. 2011). In the context of hybrid populations, assuming that greater genetic distance between parents promotes phenological divergence in their offspring, we found a positive correlation between parental flowering time differences and hybrid stand biomass. Additionally, stand biomass was maximized when flowering individuals comprised approximately 50% of the stand, suggesting that variation in phenological stages can optimize resource use and enhance hybrid stand performance. While flowering time differences showed the strongest relationship with biomass, our findings indicate that multiple traits contribute to this effect, reinforcing the idea that diverse functional strategies within a stand promote coexistence and improve overall performance. These results align with ecological theories on diversity–productivity relationships, where greater functional diversity enhances community‐level biomass through niche differentiation and reduced competition. Therefore, while individual hybrid biomass follows a hump‐shaped relationship with genetic distance likely reflecting trade‐offs between heterosis and genetic incompatibility, stand‐level biomass continues to increase with genetic distance, likely due to the accumulation of beneficial trait variation that optimizes collective performance. This highlights the importance of considering both individual and group‐level processes when assessing hybrid population success.

Subsequently, we showed that individual hybrid performance is highest in hybrids formed from combinations of different groups within the same non‐relict lineage, exhibiting moderate genetic distance, which supports the ‘optimal mating distance’ hypothesis. However, stand‐level performance peaks in hybrids with high genetic distance, formed between relict and non‐relict lineages, which are hybrids that have been observed to persist in the wild (Exposito‐Alonso et al. 2018; François et al. 2008). Remarkably, when comparing these hybrids to other types of crosses, we observed a clear contrast, emphasizing the significant advantage of this specific lineage combination in enhancing performance at the stand level. Thus, these hybrids exhibit a unique ecological compatibility that may play a crucial role in their persistence and adaptability over time. Accordingly, Campbell and Snow (Campbell and Snow 2007) emphasized the importance of stand‐level performance, suggesting that factors promoting hybrid persistence in natural populations, such as hybrid relative fitness, could enhance long‐term hybrid success. From an agronomic perspective, biomass production is fundamental, as it underpins plant productivity and resilience, particularly under high‐density conditions where natural selection often favors maximizing community‐level biomass (Weiner and Freckleton 2010). Scale‐dependent differences in biomass, as observed here, highlight the divergence between traits that benefit individual versus group performance, echoing recent findings in evolutionary agroecology (Anten and Vermeulen 2016; Golan et al. 2023; Weiner et al. 2017). These insights suggest that hybrids with optimal lineage combinations could not only enhance biomass production in crops but also serve as a model for designing breeding systems that balance individual and group performance.

## 5 Conclusion

Our findings highlight the complex interplay between genetic distance and hybrid performance at different scales. Hybrids with moderate genetic distance exhibited optimum performance at the individual level, while those with high genetic divergence demonstrated superior stand‐level biomass. This duality emphasizes the importance of scale in assessing hybrid performance and offers a framework for understanding how ecological and evolutionary dynamics shape hybrid populations. To fully understand the success and persistence of hybrid populations, we must move beyond predictions based solely on individual fitness. Hybridization is also an ecological process, where plant–plant interactions and the combination of individual traits, influencing competition or cooperation, collectively determine hybrid population success. While these results provide valuable insights into biomass‐based performance, further research is needed to directly assess the roles of cooperation and competition, examine broader fitness‐related traits, and explore environmental variability. Future long‐term, multi‐generational studies will be key to testing how multilevel selection shapes the persistence and dynamics of natural hybrid populations. Identifying lineage combinations that enhance group‐level performance may also inform breeding strategies aimed at improving stand‐level productivity and resilience.

## Conflicts of Interest

The authors declare no conflicts of interest.

## Supporting information


**Data S1:** Dataset_utami_etal

**Data S2:** Supporting Information

## Data Availability

Data on hybrid parents, hybrid biomass, and statistical analysis results are available in the supplementary section.
