# Supplementary material for: From Individual to Stand Performance in Hybrids: Challenging the Optimal Parental Genetic Distance
Source: Evol Appl. 2025 Oct 9;18(10):e70165. doi: 10.1111/eva.70165 (PMC12508626; doi:10.1111/eva.70165)
Supplement: Supplementary file 2 — Data S2: Supporting Information [file EVA-18-e70165-s002.docx]

**SUPPLEMENTARY**

**Table S1.** Information on *A.thaliana* 63 hybrids used in the study (parental ID and admixture group are based on 1001 Genomes project <https://1001genomes.org/accessions.html>)

| **Cross ID** | **Mother ID** | **Mother admixture group** | **Father ID** | **Father admixture group** | **Genetic distance** |
| --- | --- | --- | --- | --- | --- |
| hd311 | 9947 | relict | 10002 | western_europe | 0.04337757 |
| hd324 | 9947 | relict | 9997 | central_europe | 0.04415438 |
| rd004 | 7177 | central_europe | 7186 | central_europe | 0.028194316 |
| rd051 | 10018 | central_europe | 9968 | italy_balkan_caucasus | 0.023284947 |
| rd081 | 6970 | spain | 6979 | central_europe | 0.028404646 |
| rd095 | 7255 | germany | 7165 | germany | 0.026908925 |
| rd100 | 6180 | germany | 7028 | admixed | 0.025129398 |
| rd111 | 7028 | admixed | 88 | western_europe | 0.023901574 |
| rd115 | 7025 | central_europe | 7092 | western_europe | 0.029105913 |
| rd118 | 7031 | germany | 7092 | western_europe | 0.027526023 |
| rd119 | 7062 | germany | 7127 | admixed | 0.029103998 |
| rd126 | 9507 | spain | 6903 | central_europe | 0.02245638 |
| rd136 | 7396 | central_europe | 7063 | relict | 0.040060736 |
| rd138 | 9057 | south_sweden | 9920 | germany | 0.028877268 |
| rd153 | 7322 | germany | 9507 | spain | 0.021350848 |
| rd158 | 6150 | south_sweden | 9796 | central_europe | 0.028744295 |
| rd159 | 9510 | spain | 6390 | central_europe | 0.029175786 |
| rd171 | 9776 | central_europe | 6023 | south_sweden | 0.028580295 |
| rd185 | 9920 | germany | 6901 | north_sweden | 0.031468485 |
| rd199 | 159 | western_europe | 6975 | central_europe | 0.026946539 |
| rd200 | 139 | western_europe | 7288 | admixed | 0.02591848 |
| rd205 | 5907 | central_europe | 7177 | central_europe | 0.024404623 |
| rd225 | 9659 | italy_balkan_caucasus | 8376 | north_sweden | 0.030826883 |
| rd227 | 9793 | central_europe | 9537 | spain | 0.030183308 |
| rd256 | 9598 | relict | 9741 | central_europe | 0.04821588 |
| rd275 | 7063 | relict | 9394 | admixed | 0.041124105 |
| rd279 | 9794 | central_europe | 9386 | north_sweden | 0.033574987 |
| rd282 | 9988 | italy_balkan_caucasus | 6177 | north_sweden | 0.034369685 |
| rd302 | 9966 | italy_balkan_caucasus | 992 | south_sweden | 0.02484387 |
| rd307 | 6951 | central_europe | 9574 | relict | 0.037369363 |
| rd321 | 6915 | germany | 9600 | relict | 0.051899344 |
| rd323 | 6009 | north_sweden | 7164 | south_sweden | 0.032239858 |
| rd331 | 9535 | spain | 9914 | central_europe | 0.035282213 |
| rd332 | 6151 | south_sweden | 8422 | south_sweden | 0.029274728 |
| rd349 | 6216 | north_sweden | 9657 | italy_balkan_caucasus | 0.0339723 |
| rd359 | 9770 | central_europe | 6154 | north_sweden | 0.032708433 |
| rd380 | 6023 | south_sweden | 6025 | north_sweden | 0.031395618 |
| rd389 | 6241 | north_sweden | 6177 | north_sweden | 0.021080315 |
| rd390 | 1066 | south_sweden | 6035 | south_sweden | 0.024002073 |
| rd396 | 6088 | south_sweden | 9776 | central_europe | 0.029606674 |
| rd403 | 8422 | south_sweden | 9815 | central_europe | 0.028389597 |
| rd405 | 8369 | south_sweden | 6209 | north_sweden | 0.030540442 |
| rd406 | 8369 | south_sweden | 8376 | north_sweden | 0.029139034 |
| rd416 | 6069 | north_sweden | 992 | south_sweden | 0.02914054 |
| rd421 | 6071 | north_sweden | 6040 | south_sweden | 0.030825404 |
| rd425 | 9726 | italy_balkan_caucasus | 9528 | spain | 0.03743618 |
| rd437 | 6974 | south_sweden | 9584 | spain | 0.029512862 |
| rd454 | 9395 | south_sweden | 7106 | central_europe | 0.029691933 |
| rd455 | 7002 | germany | 9800 | central_europe | 0.029212026 |
| rd467 | 9433 | north_sweden | 9102 | italy_balkan_caucasus | 0.03691135 |
| rd476 | 9542 | relict | 9568 | spain | 0.051637933 |
| rd481 | 6915 | germany | 10018 | central_europe | 0.026430957 |
| rd482 | 9546 | spain | 9606 | relict | 0.03568485 |
| rd483 | 9528 | spain | 6043 | north_sweden | 0.038055386 |
| rd487 | 6177 | north_sweden | 9932 | admixed | 0.030605212 |
| rd496 | 8376 | north_sweden | 9433 | north_sweden | 0.02212154 |
| rd500 | 9528 | spain | 7002 | germany | 0.03825089 |
| rd505 | 9597 | spain | 9528 | spain | 0.036842916 |
| rd507 | 9653 | italy_balkan_caucasus | 9968 | italy_balkan_caucasus | 0.020840999 |
| rd517 | 7125 | germany | 6043 | north_sweden | 0.03208773 |
| rd550 | 9943 | spain | 9966 | italy_balkan_caucasus | 0.027957 |
| rd551 | 10002 | western_europe | 9966 | italy_balkan_caucasus | 0.024583554 |
| rd554 | 9947 | relict | 9998 | admixed | 0.04230591 |

**Table S2.** Correlation of trait variation with parental trait distances

| Pearson correlation | Parental trait difference | |
| --- | --- | --- |
| Trait variance | R2 | p-value |
| Biomass | 0.0729 | 0.03 |
| Flowering proportion | 0.1089 | 0.007 |


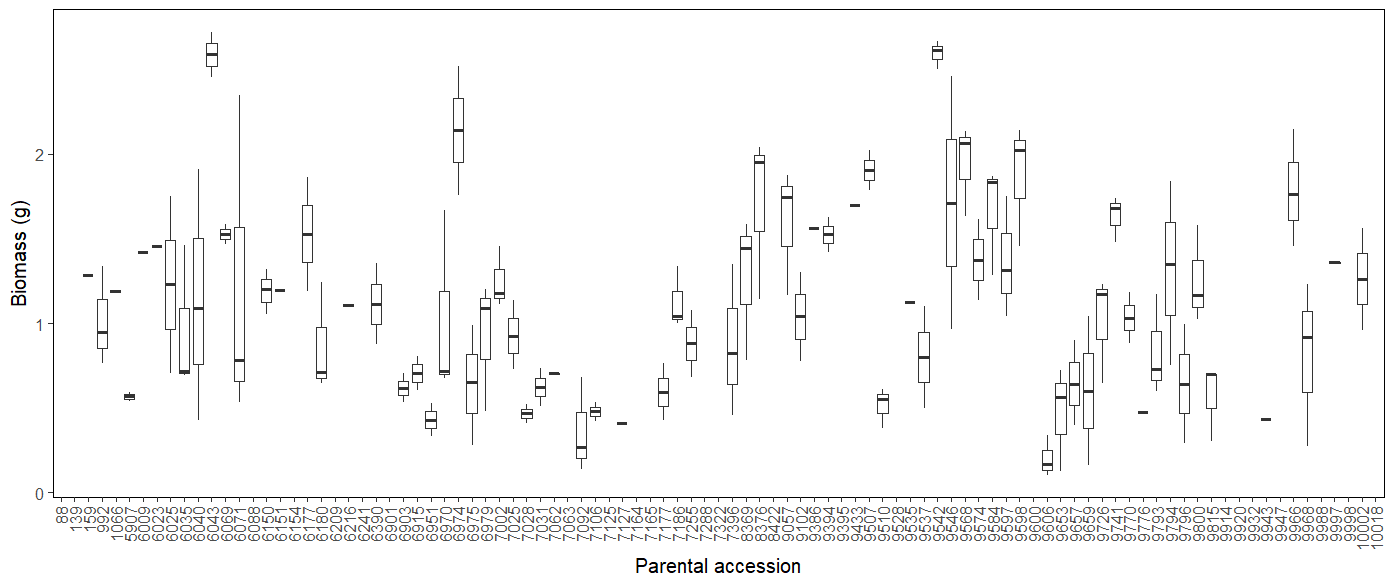


**Figure S1.** Variation of vegetative dry biomass among 97 natural *A. thaliana* accessions


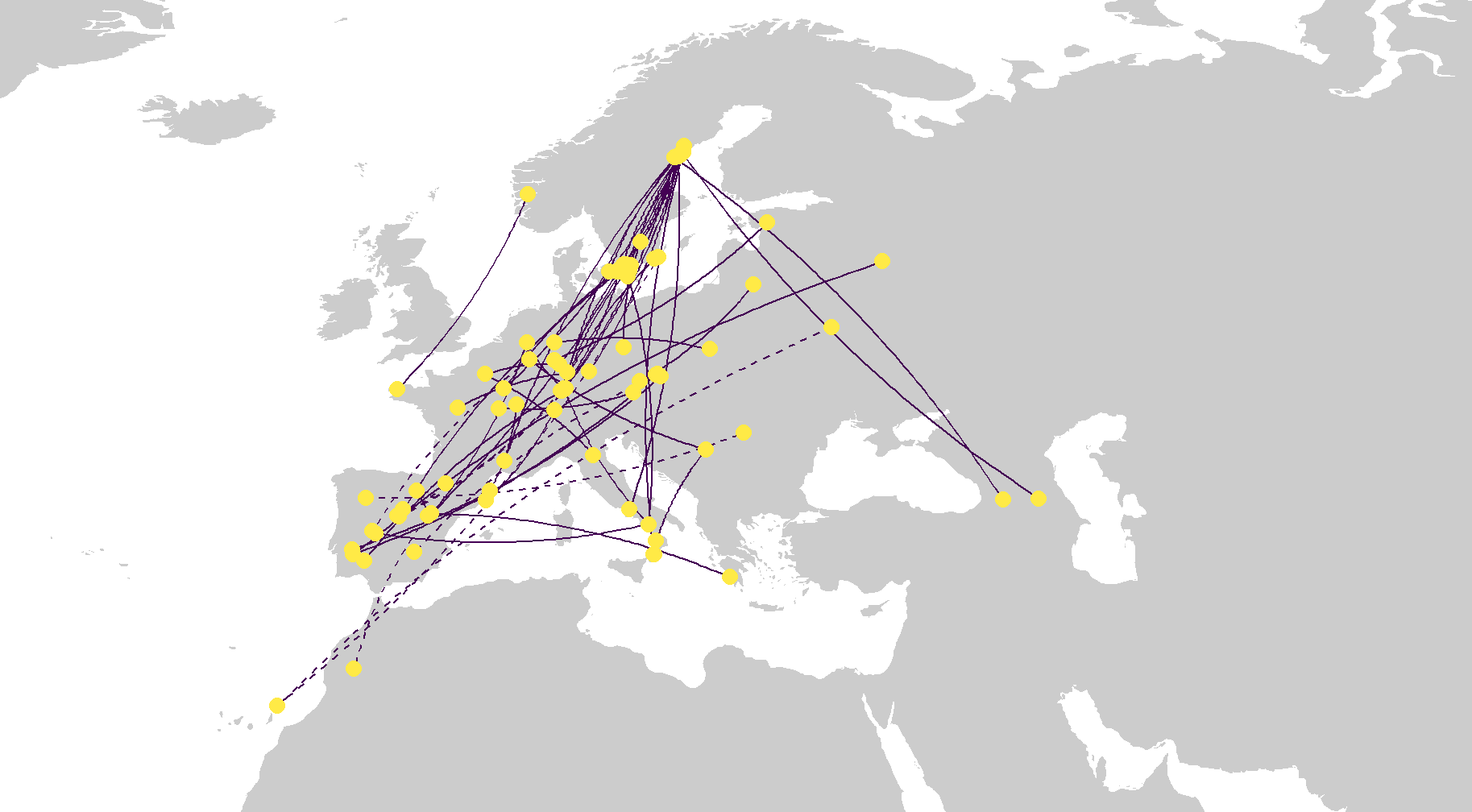


**Figure S2. Origins of parental accessions and F2 hybrid combinations in *Arabidopsis thaliana****.* Yellow points indicate the geographic origins of the 97 natural accessions used as parental lines. Purple lines connect parental pairs, representing the 63 F2 hybrid populations derived from these crosses. Dashed lines indicate pairings between relict and non-relict lineages, as defined by the 1001 Genomes Consortium (2016).

**Figure S3.** Experimental settings of the F2 hybrids populations in the green house.
